# 1-Diphenyl­methyl-4-[3-(4-fluoro­benzo­yl)prop­yl]piperazine-1,4-diium dichloride monohydrate

**DOI:** 10.1107/S1600536811037378

**Published:** 2011-09-30

**Authors:** Jing Wang, Yongli Wang, Caiqin Yang

**Affiliations:** a School of Pharmaceutical Sciences, Hebei Medical University, Shijiazhuang 050017, People’s Republic of China

## Abstract

In the title compound, C_27_H_31_FN_2_O^2+^·2Cl^−^·H_2_O, the piperazine ring adopts a chair conformation and both N atoms are protonated. The Cl^−^ anions form strong hydrogen bonds to these protons. O/N—H⋯Cl and C—H⋯O hydrogen bonds link the anions, cations and water of hydration into a three-dimensional network.

## Related literature

For a related structure, see: Zhou & Jin (1986 ▶). For the synthesis of 1-diphenyl­methyl-4-[3-(4-fluoro­benzo­yl)prop­yl]piperazine, see: Wang *et al.* (2003 ▶).
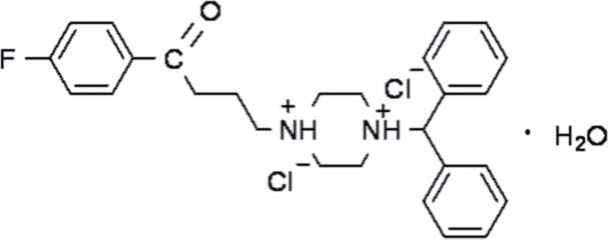


## Experimental

### 

#### Crystal data

C_27_H_31_FN_2_O^2+^·2Cl^−^·H_2_O
*M* *_r_* = 507.45
Monoclinic, 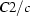

*a* = 39.2849 (14) Å
*b* = 7.3369 (3) Å
*c* = 19.5158 (7) Å
β = 107.773 (2)°
*V* = 5356.6 (3) Å^3^
*Z* = 8
Mo *K*α radiation
μ = 0.28 mm^−1^
*T* = 298 K
0.37 × 0.21 × 0.11 mm

#### Data collection

Siemens SMART CCD area-detector diffractometer
Absorption correction: multi-scan (*SADABS*; Sheldrick, 1996 ▶) *T*_min_ = 0.933, *T*_max_ = 0.970
19739 measured reflections
4566 independent reflections
3644 reflections with *I* > 2σ(*I*)
*R*_int_ = 0.034

#### Refinement

*R*[*F* ^2^ > 2σ(*F* ^2^)] = 0.046
*wR*(*F* ^2^) = 0.138
*S* = 1.03
4566 reflections
313 parameters
3 restraints
H atoms treated by a mixture of independent and constrained refinement
Δρ_max_ = 0.31 e Å^−3^
Δρ_min_ = −0.37 e Å^−3^

### 

Data collection: *SMART* (Siemens, 1994 ▶); cell refinement: *SAINT* (Siemens, 1994 ▶); data reduction: *SAINT*; program(s) used to solve structure: *SHELXS97* (Sheldrick, 2008 ▶); program(s) used to refine structure: *SHELXL97* (Sheldrick, 2008 ▶); molecular graphics: *SHELXTL* (Sheldrick, 2008 ▶); software used to prepare material for publication: *SHELXTL*.

## Supplementary Material

Crystal structure: contains datablock(s) global. DOI: 10.1107/S1600536811037378/pv2438sup1.cif

Supplementary material file. DOI: 10.1107/S1600536811037378/pv2438globalsup2.cml

Additional supplementary materials:  crystallographic information; 3D view; checkCIF report

## Figures and Tables

**Table 1:** Hydrogen-bond geometry (Å, °)

*D*—H⋯*A*	*D*—H	H⋯*A*	*D*⋯*A*	*D*—H⋯*A*
O1*W*—H1⋯Cl1^i^	1.01 (2)	2.24 (2)	3.248 (3)	176 (2)
C6—H6*A*⋯O1*W*^ii^	0.93	2.55	3.397 (4)	152
C14—H14*B*⋯O1^iii^	0.97	2.38	3.148 (3)	136
N1—H1*A*⋯Cl2	0.91	2.09	2.990 (2)	171
O1*W*—H2⋯Cl2	1.01 (2)	2.20 (2)	3.207 (3)	172 (2)
N2—H2*B*⋯Cl1	0.91	2.18	3.070 (2)	167

Symmetry codes: (i) 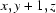; (ii) 
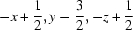
; (iii) 
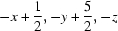
.

**Fig. 1.**
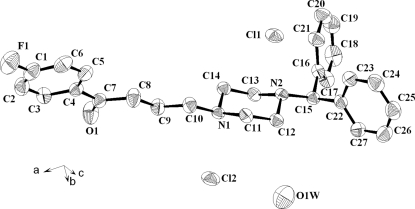
A view of the title compound, showing the atom-labelling scheme. Displacement ellipsoids are drawn at the 35% probability level.

**Fig. 2.**
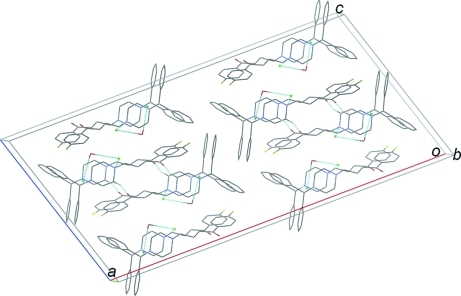
Packing diagram of the title compound showing hydrogen bonds with dotted lines.

## supplementary crystallographic information

### Comment

The crystal structure of 1-diphenylmethyl-4-[3-(4-fluorobenzoyl)propyl]piperazine has been reported (Zhou & Jin, 1986). In this article we report the structure of its dihydrochloride monohydrate.

In the title compound (Fig. 1), the piperazine ring adopts a chair conformation with the piperizine-N atoms protonated. The Cl^-^ anions form strong halogen hydrogen bonds to these protons. Two chlorine ions and one hydrone bridge piperazine cations through the O—H···Cl and N—H···Cl halogen hydrogen bonds result in a one-dimensional chain structure. Moreover, these hydrogen bonds, as well as C═O···H hydrogen bonds (Table 1), link the molecular moieties into a two dimensional sheet in the *b-c* plane. The water of hydration further consolidates the structure via hydrogen bonds of the type O—H···C. Overall, the individual molecule packs together into a three-dimensional network with a spiral structure motif (Fig. 2).

### Experimental

The 1-diphenylmethyl-4-[3-(4-fluorobenzoyl)propyl]piperazine base was synthesized according to a reported procedure (Wang *et al.*, 2003). The title compound was prepared by passing dry hydrochloride gas (100 mg) through a solution of 200 mg base in ethanol (2 ml). The single-crystals of the title compound suitable for X-ray analysis were obtained by vapor diffusion in a solution of chloroform in which the compound was soluble by benzene acting as anti-solvent.

### Refinement

The H atoms were placed at calculated positions in the riding model approximation with N—H = 0.91 Å and C—H = 0.93, 0.97 and 0.98 Å, for aryl, methylene and methyne type H-atoms, respectively, with *U*_iso_(H) = 1.2 *U*_eq_(C/N). H atoms of water molecule were located in difference Fourier maps and were refined freely with isotropic displacement parameters.

### Crystal data

**Table:**

C_27_H_31_FN_2_O^2+^·2Cl^−^·H_2_O	*F*(000) = 2144
*M**_r_* = 507.45	*D*_x_ = 1.258 Mg m^−^^3^
Monoclinic, *C*2/*c*	Mo *K*α radiation, λ = 0.71070 Å
Hall symbol: -C 2yc	Cell parameters from 6877 reflections
*a* = 39.2849 (14) Å	θ = 4.6–65.2°
*b* = 7.3369 (3) Å	µ = 0.28 mm^−^^1^
*c* = 19.5158 (7) Å	*T* = 298 K
β = 107.773 (2)°	Prismatic, colorless
*V* = 5356.6 (3) Å^3^	0.37 × 0.21 × 0.11 mm
*Z* = 8	

### Data collection

**Table:**

Siemens SMART CCD area-detector diffractometer	4566 independent reflections
Radiation source: fine-focus sealed tube	3644 reflections with *I* > 2σ(*I*)
graphite	*R*_int_ = 0.034
φ and ω scans	θ_max_ = 24.9°, θ_min_ = 1.1°
Absorption correction: multi-scan (*SADABS*; Sheldrick, 1996)	*h* = −46→44
*T*_min_ = 0.933, *T*_max_ = 0.970	*k* = −8→7
19739 measured reflections	*l* = −22→23

### Refinement

**Table:**

Refinement on *F*^2^	Primary atom site location: structure-invariant direct methods
Least-squares matrix: full	Secondary atom site location: difference Fourier map
*R*[*F*^2^ > 2σ(*F*^2^)] = 0.046	Hydrogen site location: inferred from neighbouring sites
*wR*(*F*^2^) = 0.138	H atoms treated by a mixture of independent and constrained refinement
*S* = 1.03	*w* = 1/[σ^2^(*F*_o_^2^) + (0.0742*P*)^2^ + 3.1528*P*] where *P* = (*F*_o_^2^ + 2*F*_c_^2^)/3
4566 reflections	(Δ/σ)_max_ < 0.001
313 parameters	Δρ_max_ = 0.31 e Å^−^^3^
3 restraints	Δρ_min_ = −0.37 e Å^−^^3^

### Special details

**Table:**

Geometry. All e.s.d.'s (except the e.s.d. in the dihedral angle between two l.s. planes) are estimated using the full covariance matrix. The cell e.s.d.'s are taken into account individually in the estimation of e.s.d.'s in distances, angles and torsion angles; correlations between e.s.d.'s in cell parameters are only used when they are defined by crystal symmetry. An approximate (isotropic) treatment of cell e.s.d.'s is used for estimating e.s.d.'s involving l.s. planes.
Refinement. Refinement of *F*^2^ against ALL reflections. The weighted *R*-factor *wR* and goodness of fit *S* are based on *F*^2^, conventional *R*-factors *R* are based on *F*, with *F* set to zero for negative *F*^2^. The threshold expression of *F*^2^ > σ(*F*^2^) is used only for calculating *R*-factors(gt) *etc*. and is not relevant to the choice of reflections for refinement. *R*-factors based on *F*^2^ are statistically about twice as large as those based on *F*, and *R*- factors based on ALL data will be even larger.

### Fractional atomic coordinates and isotropic or equivalent isotropic displacement parameters (Å^2^)

**Table:**

	*x*	*y*	*z*	*U*_iso_*/*U*_eq_	
F1	0.40283 (5)	0.3181 (3)	0.13299 (11)	0.1153 (7)	
Cl1	0.113600 (17)	0.81655 (8)	0.10513 (4)	0.0702 (2)	
Cl2	0.231844 (18)	1.53814 (9)	0.16249 (4)	0.0760 (2)	
N1	0.19895 (4)	1.1685 (2)	0.12880 (9)	0.0468 (4)	
H1A	0.2087	1.2821	0.1339	0.056*	
N2	0.12309 (4)	1.2321 (2)	0.10926 (8)	0.0441 (4)	
H2B	0.1166	1.1127	0.1041	0.053*	
O1	0.31404 (6)	1.0374 (3)	0.04357 (12)	0.0918 (7)	
O1W	0.17734 (9)	1.6776 (4)	0.24451 (15)	0.1295 (9)	
C1	0.37946 (8)	0.4599 (4)	0.11920 (14)	0.0765 (8)	
C2	0.38710 (7)	0.6077 (5)	0.08508 (15)	0.0792 (8)	
H2A	0.4078	0.6117	0.0714	0.095*	
C3	0.36369 (7)	0.7509 (4)	0.07114 (13)	0.0674 (6)	
H3A	0.3688	0.8540	0.0482	0.081*	
C4	0.33248 (6)	0.7454 (3)	0.09058 (11)	0.0541 (5)	
C5	0.32535 (7)	0.5884 (3)	0.12420 (12)	0.0643 (6)	
H5A	0.3044	0.5807	0.1368	0.077*	
C6	0.34905 (8)	0.4448 (4)	0.13884 (14)	0.0771 (8)	
H6A	0.3445	0.3403	0.1615	0.092*	
C7	0.30841 (6)	0.9070 (3)	0.07606 (12)	0.0572 (5)	
C8	0.27859 (7)	0.9051 (4)	0.10829 (17)	0.0789 (8)	
H8A	0.2890	0.9003	0.1602	0.095*	
H8B	0.2652	0.7931	0.0936	0.095*	
C9	0.25220 (6)	1.0625 (3)	0.09051 (14)	0.0641 (6)	
H9A	0.2646	1.1783	0.1017	0.077*	
H9B	0.2385	1.0613	0.0399	0.077*	
C10	0.22818 (6)	1.0325 (3)	0.13699 (13)	0.0596 (6)	
H10A	0.2175	0.9126	0.1263	0.072*	
H10B	0.2429	1.0319	0.1869	0.072*	
C11	0.18253 (5)	1.1380 (3)	0.18779 (11)	0.0537 (5)	
H11A	0.2003	1.1619	0.2337	0.064*	
H11B	0.1754	1.0113	0.1873	0.064*	
C12	0.15059 (5)	1.2567 (3)	0.18091 (11)	0.0541 (5)	
H12A	0.1402	1.2264	0.2187	0.065*	
H12B	0.1580	1.3833	0.1868	0.065*	
C13	0.14022 (5)	1.2799 (3)	0.05275 (11)	0.0502 (5)	
H13A	0.1484	1.4053	0.0591	0.060*	
H13B	0.1227	1.2691	0.0055	0.060*	
C14	0.17127 (5)	1.1568 (3)	0.05701 (11)	0.0506 (5)	
H14A	0.1629	1.0321	0.0482	0.061*	
H14B	0.1818	1.1905	0.0199	0.061*	
C15	0.08987 (5)	1.3469 (3)	0.10174 (11)	0.0491 (5)	
H15A	0.0973	1.4748	0.1041	0.059*	
C16	0.06201 (5)	1.3197 (3)	0.02871 (11)	0.0519 (5)	
C17	0.04723 (6)	1.4723 (4)	−0.01065 (13)	0.0651 (6)	
H17A	0.0555	1.5876	0.0063	0.078*	
C18	0.02014 (7)	1.4545 (5)	−0.07510 (15)	0.0835 (9)	
H18A	0.0100	1.5580	−0.1008	0.100*	
C19	0.00813 (7)	1.2854 (5)	−0.10126 (14)	0.0836 (9)	
H19A	−0.0099	1.2742	−0.1449	0.100*	
C20	0.02270 (7)	1.1335 (4)	−0.06319 (14)	0.0786 (8)	
H20A	0.0146	1.0187	−0.0810	0.094*	
C21	0.04927 (6)	1.1494 (4)	0.00130 (13)	0.0667 (6)	
H21A	0.0589	1.0450	0.0270	0.080*	
C22	0.07512 (5)	1.3157 (3)	0.16414 (11)	0.0517 (5)	
C23	0.06211 (7)	1.1488 (4)	0.17795 (13)	0.0659 (6)	
H23A	0.0631	1.0490	0.1493	0.079*	
C24	0.04770 (8)	1.1286 (5)	0.23378 (15)	0.0806 (8)	
H24A	0.0392	1.0157	0.2427	0.097*	
C25	0.04600 (8)	1.2757 (5)	0.27604 (15)	0.0833 (9)	
H25A	0.0360	1.2629	0.3133	0.100*	
C26	0.05902 (8)	1.4419 (5)	0.26328 (15)	0.0822 (8)	
H26A	0.0579	1.5415	0.2920	0.099*	
C27	0.07369 (6)	1.4609 (4)	0.20780 (13)	0.0645 (6)	
H27A	0.0827	1.5733	0.1998	0.077*	
H2	0.1952 (5)	1.647 (4)	0.2183 (13)	0.080*	
H1	0.1574 (5)	1.726 (4)	0.2022 (12)	0.080*	

### Atomic displacement parameters (Å^2^)

**Table:**

	*U*^11^	*U*^22^	*U*^33^	*U*^12^	*U*^13^	*U*^23^
F1	0.1138 (14)	0.1047 (15)	0.1215 (14)	0.0581 (12)	0.0270 (12)	0.0098 (11)
Cl1	0.0742 (4)	0.0398 (4)	0.0921 (5)	−0.0111 (3)	0.0189 (3)	0.0043 (3)
Cl2	0.0754 (4)	0.0481 (4)	0.1010 (5)	−0.0194 (3)	0.0220 (3)	−0.0042 (3)
N1	0.0441 (9)	0.0382 (10)	0.0579 (9)	−0.0057 (7)	0.0152 (7)	0.0007 (7)
N2	0.0423 (8)	0.0386 (9)	0.0499 (9)	−0.0064 (7)	0.0121 (7)	0.0007 (7)
O1	0.1009 (14)	0.0751 (13)	0.1225 (16)	0.0187 (11)	0.0686 (13)	0.0393 (12)
O1W	0.144 (2)	0.123 (2)	0.125 (2)	0.0220 (19)	0.0457 (18)	0.0110 (17)
C1	0.0786 (17)	0.079 (2)	0.0646 (15)	0.0263 (15)	0.0118 (13)	−0.0046 (13)
C2	0.0598 (14)	0.098 (2)	0.0800 (17)	0.0155 (15)	0.0208 (13)	−0.0043 (16)
C3	0.0631 (14)	0.0712 (17)	0.0712 (15)	0.0020 (13)	0.0254 (12)	0.0036 (12)
C4	0.0599 (12)	0.0560 (14)	0.0480 (11)	0.0000 (10)	0.0188 (9)	−0.0041 (10)
C5	0.0777 (15)	0.0558 (15)	0.0665 (14)	0.0032 (12)	0.0326 (12)	−0.0030 (11)
C6	0.109 (2)	0.0569 (16)	0.0660 (15)	0.0144 (15)	0.0272 (15)	0.0042 (12)
C7	0.0627 (13)	0.0557 (14)	0.0581 (12)	−0.0010 (11)	0.0255 (10)	0.0012 (11)
C8	0.0805 (17)	0.0615 (17)	0.111 (2)	0.0126 (14)	0.0531 (16)	0.0169 (15)
C9	0.0617 (13)	0.0576 (15)	0.0790 (15)	0.0048 (11)	0.0304 (12)	0.0067 (12)
C10	0.0559 (12)	0.0500 (14)	0.0749 (14)	0.0084 (10)	0.0229 (11)	0.0085 (11)
C11	0.0470 (11)	0.0611 (14)	0.0518 (11)	−0.0029 (10)	0.0134 (9)	0.0033 (10)
C12	0.0461 (11)	0.0627 (14)	0.0508 (11)	−0.0054 (10)	0.0108 (9)	−0.0042 (10)
C13	0.0486 (11)	0.0508 (13)	0.0513 (11)	−0.0047 (9)	0.0155 (9)	0.0072 (9)
C14	0.0520 (11)	0.0502 (13)	0.0511 (11)	−0.0057 (9)	0.0179 (9)	0.0006 (9)
C15	0.0452 (11)	0.0420 (12)	0.0593 (12)	−0.0013 (9)	0.0150 (9)	0.0017 (9)
C16	0.0440 (11)	0.0548 (14)	0.0586 (12)	−0.0001 (9)	0.0182 (9)	0.0064 (10)
C17	0.0606 (13)	0.0635 (16)	0.0716 (15)	0.0045 (11)	0.0210 (11)	0.0164 (12)
C18	0.0712 (17)	0.103 (2)	0.0725 (17)	0.0185 (16)	0.0164 (14)	0.0345 (17)
C19	0.0618 (15)	0.116 (3)	0.0626 (15)	0.0045 (17)	0.0036 (12)	0.0000 (16)
C20	0.0600 (14)	0.087 (2)	0.0766 (16)	−0.0051 (14)	0.0030 (13)	−0.0089 (15)
C21	0.0544 (13)	0.0627 (16)	0.0728 (15)	−0.0016 (11)	0.0041 (11)	0.0014 (12)
C22	0.0399 (10)	0.0559 (14)	0.0568 (12)	0.0007 (9)	0.0109 (9)	0.0013 (10)
C23	0.0673 (14)	0.0645 (16)	0.0715 (15)	−0.0109 (12)	0.0296 (12)	−0.0033 (12)
C24	0.0774 (17)	0.090 (2)	0.0803 (17)	−0.0155 (15)	0.0334 (14)	0.0072 (16)
C25	0.0710 (17)	0.120 (3)	0.0631 (15)	−0.0051 (17)	0.0271 (13)	−0.0011 (16)
C26	0.0793 (18)	0.098 (2)	0.0704 (16)	0.0046 (17)	0.0243 (14)	−0.0194 (15)
C27	0.0610 (13)	0.0642 (16)	0.0665 (14)	−0.0005 (11)	0.0167 (11)	−0.0079 (12)

### Geometric parameters (Å, °)

**Table:**

F1—C1	1.359 (3)	C11—H11B	0.9700
N1—C14	1.491 (3)	C12—H12A	0.9700
N1—C10	1.493 (3)	C12—H12B	0.9700
N1—C11	1.498 (3)	C13—C14	1.500 (3)
N1—H1A	0.9100	C13—H13A	0.9700
N2—C12	1.494 (3)	C13—H13B	0.9700
N2—C13	1.498 (2)	C14—H14A	0.9700
N2—C15	1.522 (3)	C14—H14B	0.9700
N2—H2B	0.9100	C15—C22	1.517 (3)
O1—C7	1.206 (3)	C15—C16	1.522 (3)
O1W—H2	1.012 (16)	C15—H15A	0.9800
O1W—H1	1.013 (16)	C16—C17	1.381 (3)
C1—C2	1.353 (4)	C16—C21	1.391 (3)
C1—C6	1.366 (4)	C17—C18	1.384 (4)
C2—C3	1.368 (4)	C17—H17A	0.9300
C2—H2A	0.9300	C18—C19	1.369 (4)
C3—C4	1.390 (3)	C18—H18A	0.9300
C3—H3A	0.9300	C19—C20	1.365 (4)
C4—C5	1.396 (3)	C19—H19A	0.9300
C4—C7	1.489 (3)	C20—C21	1.373 (3)
C5—C6	1.377 (4)	C20—H20A	0.9300
C5—H5A	0.9300	C21—H21A	0.9300
C6—H6A	0.9300	C22—C27	1.376 (3)
C7—C8	1.489 (3)	C22—C23	1.384 (3)
C8—C9	1.519 (4)	C23—C24	1.381 (4)
C8—H8A	0.9700	C23—H23A	0.9300
C8—H8B	0.9700	C24—C25	1.372 (4)
C9—C10	1.511 (3)	C24—H24A	0.9300
C9—H9A	0.9700	C25—C26	1.374 (4)
C9—H9B	0.9700	C25—H25A	0.9300
C10—H10A	0.9700	C26—C27	1.380 (4)
C10—H10B	0.9700	C26—H26A	0.9300
C11—C12	1.499 (3)	C27—H27A	0.9300
C11—H11A	0.9700		
			
C14—N1—C10	112.26 (17)	N2—C12—H12A	109.4
C14—N1—C11	110.60 (15)	C11—C12—H12A	109.4
C10—N1—C11	108.40 (16)	N2—C12—H12B	109.4
C14—N1—H1A	108.5	C11—C12—H12B	109.4
C10—N1—H1A	108.5	H12A—C12—H12B	108.0
C11—N1—H1A	108.5	N2—C13—C14	111.04 (16)
C12—N2—C13	107.56 (15)	N2—C13—H13A	109.4
C12—N2—C15	112.16 (16)	C14—C13—H13A	109.4
C13—N2—C15	111.36 (15)	N2—C13—H13B	109.4
C12—N2—H2B	108.6	C14—C13—H13B	109.4
C13—N2—H2B	108.6	H13A—C13—H13B	108.0
C15—N2—H2B	108.6	N1—C14—C13	111.42 (17)
H2—O1W—H1	98.6 (16)	N1—C14—H14A	109.3
C2—C1—F1	118.2 (3)	C13—C14—H14A	109.3
C2—C1—C6	123.2 (3)	N1—C14—H14B	109.3
F1—C1—C6	118.6 (3)	C13—C14—H14B	109.3
C1—C2—C3	118.5 (3)	H14A—C14—H14B	108.0
C1—C2—H2A	120.8	C22—C15—C16	112.97 (16)
C3—C2—H2A	120.8	C22—C15—N2	111.43 (16)
C2—C3—C4	121.2 (3)	C16—C15—N2	111.58 (16)
C2—C3—H3A	119.4	C22—C15—H15A	106.8
C4—C3—H3A	119.4	C16—C15—H15A	106.8
C3—C4—C5	118.2 (2)	N2—C15—H15A	106.8
C3—C4—C7	119.1 (2)	C17—C16—C21	118.3 (2)
C5—C4—C7	122.6 (2)	C17—C16—C15	118.3 (2)
C6—C5—C4	120.6 (2)	C21—C16—C15	123.32 (19)
C6—C5—H5A	119.7	C16—C17—C18	120.3 (3)
C4—C5—H5A	119.7	C16—C17—H17A	119.8
C1—C6—C5	118.3 (3)	C18—C17—H17A	119.8
C1—C6—H6A	120.9	C19—C18—C17	120.4 (3)
C5—C6—H6A	120.9	C19—C18—H18A	119.8
O1—C7—C4	121.6 (2)	C17—C18—H18A	119.8
O1—C7—C8	121.6 (2)	C20—C19—C18	119.8 (2)
C4—C7—C8	116.6 (2)	C20—C19—H19A	120.1
C7—C8—C9	117.8 (2)	C18—C19—H19A	120.1
C7—C8—H8A	107.9	C19—C20—C21	120.3 (3)
C9—C8—H8A	107.9	C19—C20—H20A	119.8
C7—C8—H8B	107.9	C21—C20—H20A	119.8
C9—C8—H8B	107.9	C20—C21—C16	120.8 (2)
H8A—C8—H8B	107.2	C20—C21—H21A	119.6
C10—C9—C8	105.03 (19)	C16—C21—H21A	119.6
C10—C9—H9A	110.7	C27—C22—C23	118.5 (2)
C8—C9—H9A	110.7	C27—C22—C15	118.5 (2)
C10—C9—H9B	110.7	C23—C22—C15	123.0 (2)
C8—C9—H9B	110.7	C24—C23—C22	120.9 (3)
H9A—C9—H9B	108.8	C24—C23—H23A	119.6
N1—C10—C9	116.04 (19)	C22—C23—H23A	119.6
N1—C10—H10A	108.3	C25—C24—C23	119.8 (3)
C9—C10—H10A	108.3	C25—C24—H24A	120.1
N1—C10—H10B	108.3	C23—C24—H24A	120.1
C9—C10—H10B	108.3	C24—C25—C26	120.0 (3)
H10A—C10—H10B	107.4	C24—C25—H25A	120.0
N1—C11—C12	112.92 (17)	C26—C25—H25A	120.0
N1—C11—H11A	109.0	C25—C26—C27	120.0 (3)
C12—C11—H11A	109.0	C25—C26—H26A	120.0
N1—C11—H11B	109.0	C27—C26—H26A	120.0
C12—C11—H11B	109.0	C22—C27—C26	120.9 (3)
H11A—C11—H11B	107.8	C22—C27—H27A	119.6
N2—C12—C11	111.11 (17)	C26—C27—H27A	119.6
			
F1—C1—C2—C3	−179.9 (2)	N2—C13—C14—N1	−59.1 (2)
C6—C1—C2—C3	1.7 (4)	C12—N2—C15—C22	−52.4 (2)
C1—C2—C3—C4	−0.8 (4)	C13—N2—C15—C22	−172.96 (17)
C2—C3—C4—C5	−0.7 (4)	C12—N2—C15—C16	−179.67 (17)
C2—C3—C4—C7	178.3 (2)	C13—N2—C15—C16	59.7 (2)
C3—C4—C5—C6	1.3 (3)	C22—C15—C16—C17	103.2 (2)
C7—C4—C5—C6	−177.7 (2)	N2—C15—C16—C17	−130.3 (2)
C2—C1—C6—C5	−1.1 (4)	C22—C15—C16—C21	−73.2 (3)
F1—C1—C6—C5	−179.5 (2)	N2—C15—C16—C21	53.3 (3)
C4—C5—C6—C1	−0.4 (4)	C21—C16—C17—C18	0.9 (3)
C3—C4—C7—O1	5.1 (4)	C15—C16—C17—C18	−175.7 (2)
C5—C4—C7—O1	−176.0 (2)	C16—C17—C18—C19	−1.2 (4)
C3—C4—C7—C8	−170.5 (2)	C17—C18—C19—C20	0.7 (5)
C5—C4—C7—C8	8.4 (3)	C18—C19—C20—C21	0.2 (5)
O1—C7—C8—C9	8.1 (4)	C19—C20—C21—C16	−0.5 (4)
C4—C7—C8—C9	−176.2 (2)	C17—C16—C21—C20	0.0 (4)
C7—C8—C9—C10	−173.9 (2)	C15—C16—C21—C20	176.4 (2)
C14—N1—C10—C9	68.0 (3)	C16—C15—C22—C27	−115.0 (2)
C11—N1—C10—C9	−169.6 (2)	N2—C15—C22—C27	118.5 (2)
C8—C9—C10—N1	−178.9 (2)	C16—C15—C22—C23	63.2 (3)
C14—N1—C11—C12	−51.4 (2)	N2—C15—C22—C23	−63.4 (3)
C10—N1—C11—C12	−174.83 (18)	C27—C22—C23—C24	0.6 (4)
C13—N2—C12—C11	−59.0 (2)	C15—C22—C23—C24	−177.5 (2)
C15—N2—C12—C11	178.27 (17)	C22—C23—C24—C25	0.4 (4)
N1—C11—C12—N2	55.8 (2)	C23—C24—C25—C26	−0.8 (4)
C12—N2—C13—C14	61.0 (2)	C24—C25—C26—C27	0.2 (4)
C15—N2—C13—C14	−175.72 (16)	C23—C22—C27—C26	−1.2 (4)
C10—N1—C14—C13	173.75 (17)	C15—C22—C27—C26	177.0 (2)
C11—N1—C14—C13	52.5 (2)	C25—C26—C27—C22	0.8 (4)

### Hydrogen-bond geometry (Å, °)

**Table:**

*D*—H···*A*	*D*—H	H···*A*	*D*···*A*	*D*—H···*A*
O1W—H1···Cl1^i^	1.01 (2)	2.24 (2)	3.248 (3)	176 (2)
C15—H15A···Cl1^i^	0.98	2.59	3.565 (2)	177
C6—H6A···O1W^ii^	0.93	2.55	3.397 (4)	152
C10—H10B···Cl2^iii^	0.97	2.80	3.746 (3)	165
C14—H14B···O1^iv^	0.97	2.38	3.148 (3)	136
N1—H1A···Cl2	0.91	2.09	2.990 (2)	171
O1W—H2···Cl2	1.01 (2)	2.20 (2)	3.207 (3)	172 (2)
N2—H2B···Cl1	0.91	2.18	3.070 (2)	167
C12—H12B···O1W	0.97	2.45	3.374 (4)	160
C21—H21A···Cl1	0.93	2.78	3.651 (3)	155

Symmetry codes: (i) *x*, *y*+1, *z*; (ii) −*x*+1/2, *y*−3/2, −*z*+1/2; (iii) −*x*+1/2, *y*−1/2, −*z*+1/2; (iv) −*x*+1/2, −*y*+5/2, −*z*.
